# Oral administration of coenzyme Q10 ameliorates memory impairment induced by nicotine-ethanol abstinence through restoration of biochemical changes in male rat hippocampal tissues

**DOI:** 10.1038/s41598-024-61932-4

**Published:** 2024-05-18

**Authors:** S. Mohammad Ahmadi-Soleimani, Seyedalireza Ghasemi, Mohamad Amin Rahmani, Moein Gharaei, Maryam Mohammadi Bezanaj, Farimah Beheshti

**Affiliations:** 1 Neuroscience Research Center, Torbat Heydariyeh University of Medical Sciences, Torbat Heydariyeh, Iran; 2 Department of Physiology, School of Medicine, Torbat Heydariyeh University of Medical Sciences, Torbat Heydariyeh, Iran; 3 Clinical Research Development Unit of Torfe Medical Center, Shahid Beheshti University of Medical Science, Tehran, Iran; 4 Student Research Committee, Torbat Heydariyeh University of Medical Sciences, Torbat Heydariyeh, Iran

**Keywords:** Adolescence, Nicotine, Ethanol, Coenzyme Q10, Abstinence, Memory, Rat, Biochemistry, Developmental biology

## Abstract

Substance abuse among adolescents has become a growing issue throughout the world. The significance of research on this life period is based on the occurrence of neurobiological changes in adolescent brain which makes the individual more susceptible for risk-taking and impulsive behaviors. Alcohol and nicotine are among the most available drugs of abuse in adolescents. Prolonged consumption of nicotine and alcohol leads to drug dependence and withdrawal which induce various dysfunctions such as memory loss. Coenzyme Q10 (CoQ_10)_ is known to improve learning and memory deficits induced by various pathological conditions such as Diabetes mellitus and Alzheimer's disease. In the present study we investigated whether CoQ_10_ treatment ameliorates memory loss following a nicotine-ethanol abstinence. Morris water maze and novel object recognition tests were done in male Wistar rats undergone nicotine-ethanol abstinence and the effect of CoQ10 was assessed on at behavioral and biochemical levels. Results indicated that nicotine-ethanol abstinence induces memory dysfunction which is associated with increased oxidative and inflammatory response, reduced cholinergic and neurotrophic function plus elevated Amyloid-B levels in hippocampi. CoQ_10_ treatment prevented memory deficits and biochemical alterations. Interestingly, this ameliorative effect of CoQ_10_ was found to be dose-dependent in most experiments and almost equipotential to that of bupropion and naloxone co-administration. CoQ_10_ treatment could effectively improve memory defects induced by nicotine-ethanol consumption through attenuation of oxidative damage, inflammation, amyloid-B level and enhancement of cholinergic and neurotrophic drive. Further studies are required to assess the unknown side effects and high dose tolerability of the drug in human subjects.

## Introduction

During the last decade, substance abuse among adolescents has become an alarming concern worldwide and this imposes a remarkable burden on global public health and health care systems^1–4^. Although there exists a rather rich literature on the biological mechanisms of drug dependence and addiction in adult individuals^5–8^, this matter has until recently remained largely underrated in the context of adolescent-specific complications. The significance of focus on this developmental period by researchers is based on the extensive evidence indicating neurobiological features of adolescent brain in humans^9,10^. These basically include structural and functional neuroadaptations such as axonal growth, myelination, synaptic pruning, and reduced volume of gray matter and changes of activity in neurotransmitter systems regulating reward-related behaviors^11–13^. Clinical studies have shown that among the wide variety of psychoactive drugs, alcohol and nicotine fall into the category of cheapest and most-easily available options for adolescents^14^. As for the nicotine, most adult smokers have reported the initiation of tobacco smoking before the age of 18^15^. A more alarming aspect of substance abuse is now the growing trend of tobacco-alcohol co-use among adolescents. This concern is based on solid evidences indicating that each of this drugs acts as a gate for the use of the other one. In other words, consumption of either alcohol or nicotine during adolescence biologically makes the individual more susceptible to show craving for the other drug^16–19^. In this regard, statistical data indicates that in the United States, adolescents initiate alcohol drinking and tobacco smoking at almost the same average age (17.0 and 17.6 years, respectively)^20^. It should be noted that during adolescence alcohol drinking is often characterized by episodes of binge consumption. Some researchers have likened the alcohol-tobacco co-use to a two-way street in which on one hand, adolescent binge drinkers are more likely to become tobacco smokers and on the other hand adolescent tobacco smokers are more likely to become binge drinkers, each compared to their control counterparts^21^.

As for the age-related outcomes, experimental evidence indicate that adolescent animals are biologically more sensitive to the mentioned rewarding effects of nicotine compared to adult subjects. Furthermore, they exhibit less aversion to nicotine intake than adults which could explain their higher desire for nicotine consumption^22–27^. In addition, a more critical aspect of chronic nicotine intake is development of nicotine dependence and the subsequent withdrawal periods following drugs cessation. Nicotine abstinence has been found to induce various deficits among which memory and learning dysfunction have widely been studied during the last ten years^28–34^. Almost the same scenario goes for prolonged ethanol effects in respect with the learning and memory function^35–39^. According to the reports of the National Institute on Drug Abuse in United States, the percentage of adolescents reporting drug abuse showed a decline in 2021 and the rate of decline in young males was found to be greater than females, leading to females now engaging in more alcohol use in adolescence. A similar trend and sex-related discrepancy has also been reported for e-cigarettes (vaping) in adolescents^40^. Experimental findings have shown that ethanol-induced memory dysfunction occurs following chronic (> 6 months) consumption and this condition is accompanied with permanent damages to various brain tissues^34,36,41–45^. As for the mechanisms underlying the mentioned detrimental effects of nicotine and ethanol abstinence, literature evidence suggest the alteration of brain oxidative damage, inflammatory response, cholinergic activity and neurotrophic factors^33,34,44,45^. Therefore, in attempt to find new therapeutic options for prevention or amelioration of nicotine/ethanol effects, consideration of these mechanism are of great significance.

Coenzyme Q10 (CoQ10) is well known as a key regulator of the oxidative phosphorylation process in mitochondria. It promotes the synthesis of ATP from carbohydrates and fatty acids inside the cell to run the cellular machinery of different pathways. In addition, CoQ10 plays a critical role in redox control of cell signaling and gene expression^46^. There are evidence indicating that CoQ10 promotes the cell growth, suppresses the apoptosis and increases the anti-oxidant defense mechanisms^46^ . In this regard, CoQ10 has widely been shown to improve learning and memory deficits induced by various factors such as Diabetes mellitus^47,48^, Parkinson's and Alzheimer’s disease^49,50^ and anesthetics^51^. The beneficial effects of CoQ_10_ on memory function were found to be mediated by reduction of oxidant markers^48^, enhancement of cholinergic transmission^48^, improvement of synaptic and mitochondrial function as well as increased concentration ATP^51^ throughput the central nervous system. In our recent studies, we observed that abstinence from each of ethanol alone^44,45^ or nicotine alone^33,52^ could impair memory function and alter biochemical parameters in rats’ brains using the same procedures/concentrations we used in this work. Therefore, in the present study we aimed to find out how abstinence from co-exposure of these drugs, as a growing social concern, might affect the results. Furthermore, the present study aimed to find out whether CoQ_10_ supplementation during adolescence could mitigate the occurrence of bio behavioral complications following nicotine-ethanol abstinence in adulthood.

## Results

### Behavioral manifestations associated with nicotine-ethanol abstinence

Results indicated that following nicotine-ethanol abstinence (PNDS 42–63), animals display specific somatic signs including tremor, eye blink, yawning and grooming, when compared to the control (vehicle-treated) group (*p* < 0.0001). In addition, the number of animals displaying theses signs per group was significantly reduced following treatment by escalating doses of CoQ10. Similar results were found in rats treated by bupropion-naloxone combination and no abnormal manifestation was observed in naïve animals treated by CoQ10 400mg/kg alone (Table 1).

**Table 1:** Expression of ethanol-nicotine withdrawal signs in different experimental groups.

Experimental group	Number of animals displaying withdrawal signs (out of 10)			
	Tremor	Eye blink	Yawning	Grooming
Vehicle	0	0	0	0
Nic-Eth	10****	9 ***	10****	8***
Nic-Eth-CoQ10 (100mg/kg)	10	9 ***	9 ***	9 ***
Nic-Eth-CoQ10 (200mg/kg)	6 *	7 **	7 **	6 *
Nic-Eth-CoQ10 (400mg/kg)	5 *	4	5 *	6 *
Nic-Eth-Bup-Nal	4	5 *	5 *	5 *
CoQ10 400mg/kg	0	0	0	0

*vs. Vehicle, *p < 0.05, **p < 0.01, ***p < 0.001, ****p < 0.0001, Data analyzed by Fisher's exact test, n = 10 in each group.

### Quantification of ethanol consumption profile and blood ethanol levels

**Figure 1:**
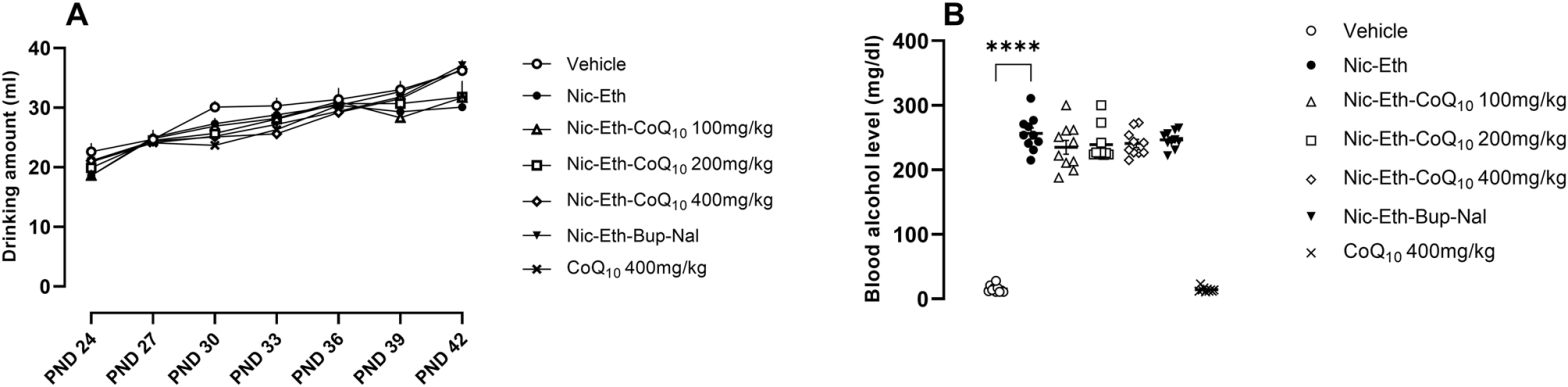
Ethanol drinking profile and blood ethanol levels. (**A**) Ethanol drinking amount (ml) was gradually increased during the entire experimental protocol in all groups (only 7 time points are shown within PNDs 21–42) and this caused a dramatic increase in blood ethanol levels (mg/dl) of all animals received ethanol in their drinking water, compared to the control group (*p* < 0.0001). Abbreviations: Nic: nicotine, Eth: ethanol, CoQ10: Coenzyme Q10, Bup: bupropion, Nal: naloxone, P*** < 0.001 vs. Vehicle.

As indicated in Fig. 1, ethanol drinking amount (ml) was gradually increased during the entire experimental protocol in all groups (part A) and this caused a dramatic increase in blood ethanol levels (mg/dl) of all animals received ethanol in their drinking water, compared to the control group (part B, p < 0.0001). Our measurements indicated that the mean blood ethanol level in these animals reached an average of 243.64 mg/dl (pooled data, n = 50, not shown), which meets the criterion of binge consumption in humans, as defined by National Institute of Health^53^.

### CoQ_10_ treatment improved the impairment of memory induced by nicotine-ethanol abstinence in MWM test

**Figure 2:**
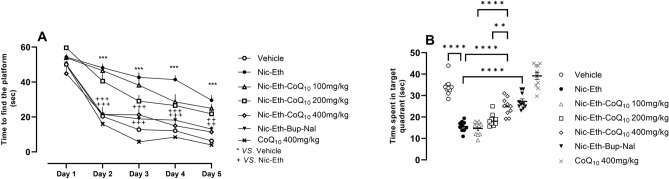
Assessment of memory by MWM test. Nic-Eth abstinence impaired the development of spatial memory in rats. This is characterized by reduced slope of the curve in Nic-Eth vs. Vehicle group. CoQ_10_ treatment, dose dependently reversed the mentioned effects of Nic-Eth abstinence (**A**). Nic-Eth group displayed reduced time of swimming in the target quadrant vs. the control subjects and this effect was reversed by CoQ_10_ treatment at the dose 400mg/kg. Furthermore, bupropion-naloxone combination (Nic-Eth-Bup-Nal group) could effectively reverse the effect of Nic-Eth abstinence almost with a potency equal to that of CoQ10 400 mg/kg. Administration of CoQ_10_ 400 mg/kg in naïve rats caused the most potent ameliorative effect in all experimental groups (**B**). Abbreviations: Nic: nicotine, Eth: ethanol, CoQ10: Coenzyme Q10, Bup: bupropion, Nal: naloxone and MWM: Morris water maze. P*** < 0.001 vs. Vehicle, *P* +  +  < 0.01 and *P* +  +  +  < 0.001 vs. Nic-Eth. P**** < 0.0001.

Our findings revealed a significant main effect for treatment (F (6, 1365) = 4.211, *P* < 0.001), day (F (4, 1365) = 6.209; *P* < 0.001) and treatment × day interaction (F (24, 1365) = 5.110, *P *< 0.05) on the escape latency to find the platform. The results of MWM revealed that Nic-Eth abstinence impairs the development of spatial memory in rats. This is characterized by reduced slope of the curve in Nic-Eth vs. Vehicle group indicating that animals undergone adolescent Nic-Eth abstinence less easily learn and remember the location of escape platform compared to their control counterparts (Fig. 2A). Interestingly, CoQ_10_ treatment, dose dependently reversed the mentioned effects of Nic-Eth abstinence (characterized by increased slope of the curve) (Fig. 2A). This finding is also consistent with reduced time of swimming in the target quadrant (platform location) in Nic-Eth group vs. the control subjects (F (6, 63) = 21.603, *P* < 0.001, Fig. 2B). CoQ_10_ at dose 400mg/kg increased time of swimming in target quadrant (*P* < 0.01; Fig. 2B). For both indices, bupropion-naloxone combination (Nic-Eth-Bup-Nal group) could effectively reverse the effect of Nic-Eth abstinence almost with a potency equal to that of CoQ_10_ 400 mg/kg (Nic-Eth-Q10 400 group) (*P* < 0.001; Fig. 2A and B). Administration of CoQ_10_ 400 mg/kg in naïve rats induced the most significant ameliorative (i.e., memory improving) effect in all experimental groups (*P* < 0.05; Fig. 2A and B). In addition, dose–response comparisons in part B revealed that the effect of CoQ_10_ increases following dose escalation (*P* < 0.0001 for 400 mg/kg vs. 100 mg/kg and *P* < 0.01 for 400 mg/kg vs. 200 mg/kg).

### CoQ10 treatment improved the impairment of memory induced by nicotine-ethanol abstinence in NOR test

**Figure 3:**
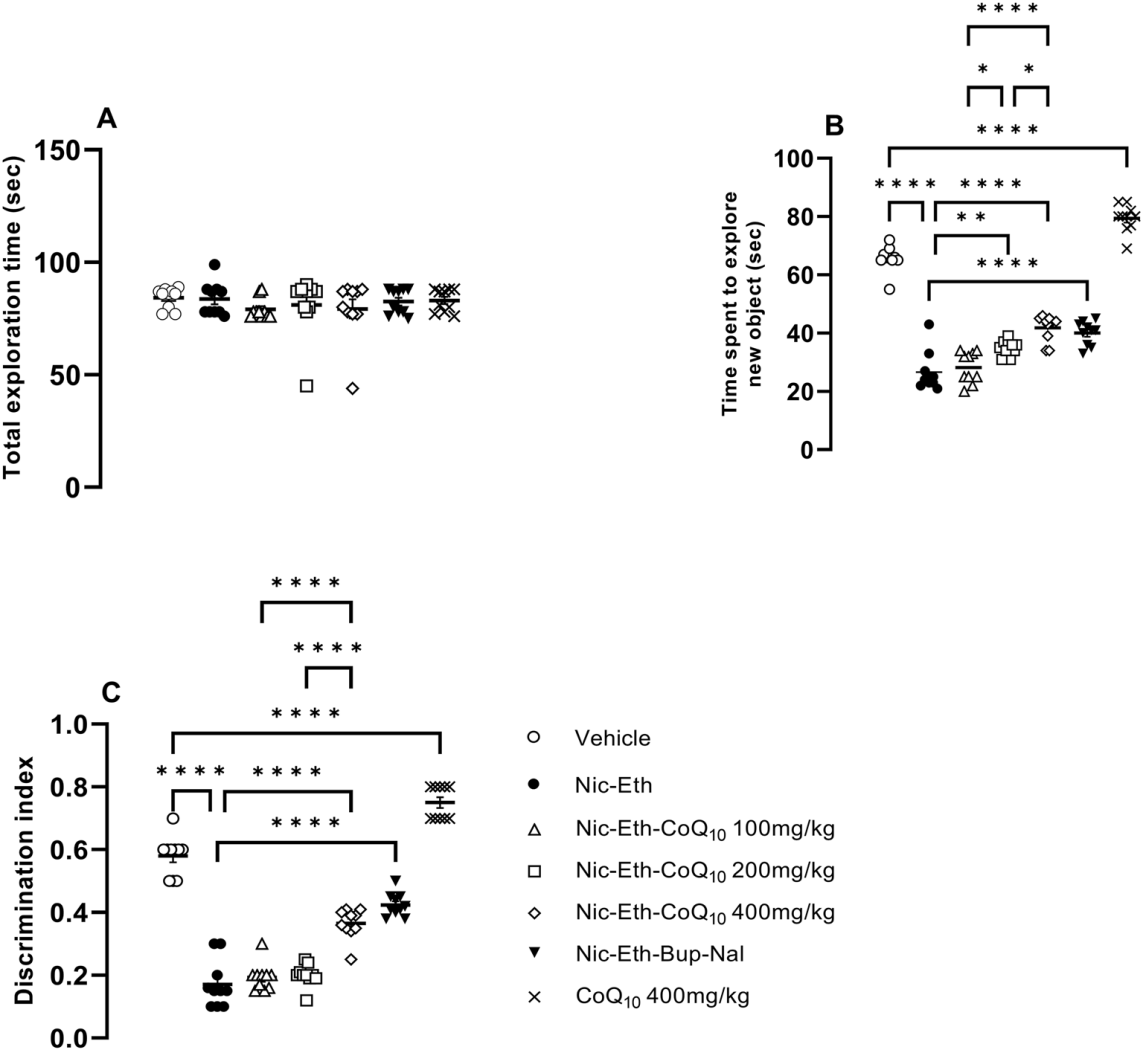
Assessment of memory by NOR test. Total exploration time was not different among the experimental groups (**A**). Animals undergone Nic-Eth abstinence, displayed impaired memory function compared to the control (vehicle) group. This was characterized by reduction in the time animal spent to explore the novel object (**B**) and the reduced discrimination index (**C**). As shown, this adverse effect was dose dependently reversed by CoQ_10_ treatment (**B** and **C**). Abbreviations: Nic: nicotine, Eth: ethanol, CoQ10: Coenzyme Q10, Bup: bupropion, Nal: naloxone and NORT: novel object recognition test. P** < 0.01 and P**** < 0.0001.

As shown, total exploration time was not different among the experimental groups (Fig. 3A), however, animals that experienced Nic-Eth abstinence during adolescence, displayed impaired memory compared to the control (vehicle) group (F (6, 63) = 16.5, *P* < 0.001, Fig. 3B). This effect was characterized by reduction in the time animal spent to explore the novel object, as well as reduction of discrimination index (F (6, 63) = 12.25, *P* < 0.001; Fig. 3C). Interestingly, this adverse effect was dose dependently improved by CoQ_10_ treatment i.e., Nic-Eth- CoQ_10_ -treated animals (200 and 400 mg/kg) spent more time to explore the novel object compared to the Nic-Eth-treated subjects (*P* < 0.05 and *P* < 0.001, respectively; Fig. 3B). This is also shown as gradual increase in discrimination index following treatment with escalating doses of CoQ10 (P < 0.05 and *P* < 0.001, respectively; Fig. 3C). Moreover, treatment with combination of bupropion and naloxone (Nic-Eth-Bup-Nal group) reversed the effect of Nic-Eth abstinence almost with an efficacy similar to that of CoQ_10_ 400 mg/kg (Nic-Eth- CoQ_10_ 400 group) (*P* < 0.001; Fig. 3B and C). The most potent effect was observed in naïve rats that only received CoQ_10_ 400 mg/kg (*P* < 0.001; Fig. 3B and C). Dose–response comparisons in part B revealed that the effect of CoQ_10_ increases following dose escalation (*P* < 0.0001 for 400 mg/kg vs. 100 mg/kg, *P* < 0.05 for 400 mg/kg vs. 200 mg/kg) and *P* < 0.05 for 200 mg/kg vs. 100 mg/kg). In part C, results were as follows: *P* < 0.0001 for 400 mg/kg vs. 100 mg/kg and *P *< 0.0001 for 400 mg/kg vs. 200 mg/kg.

### CoQ10 treatment ameliorated the oxidative profile induced by nicotine-ethanol abstinence

**Figure 4:**
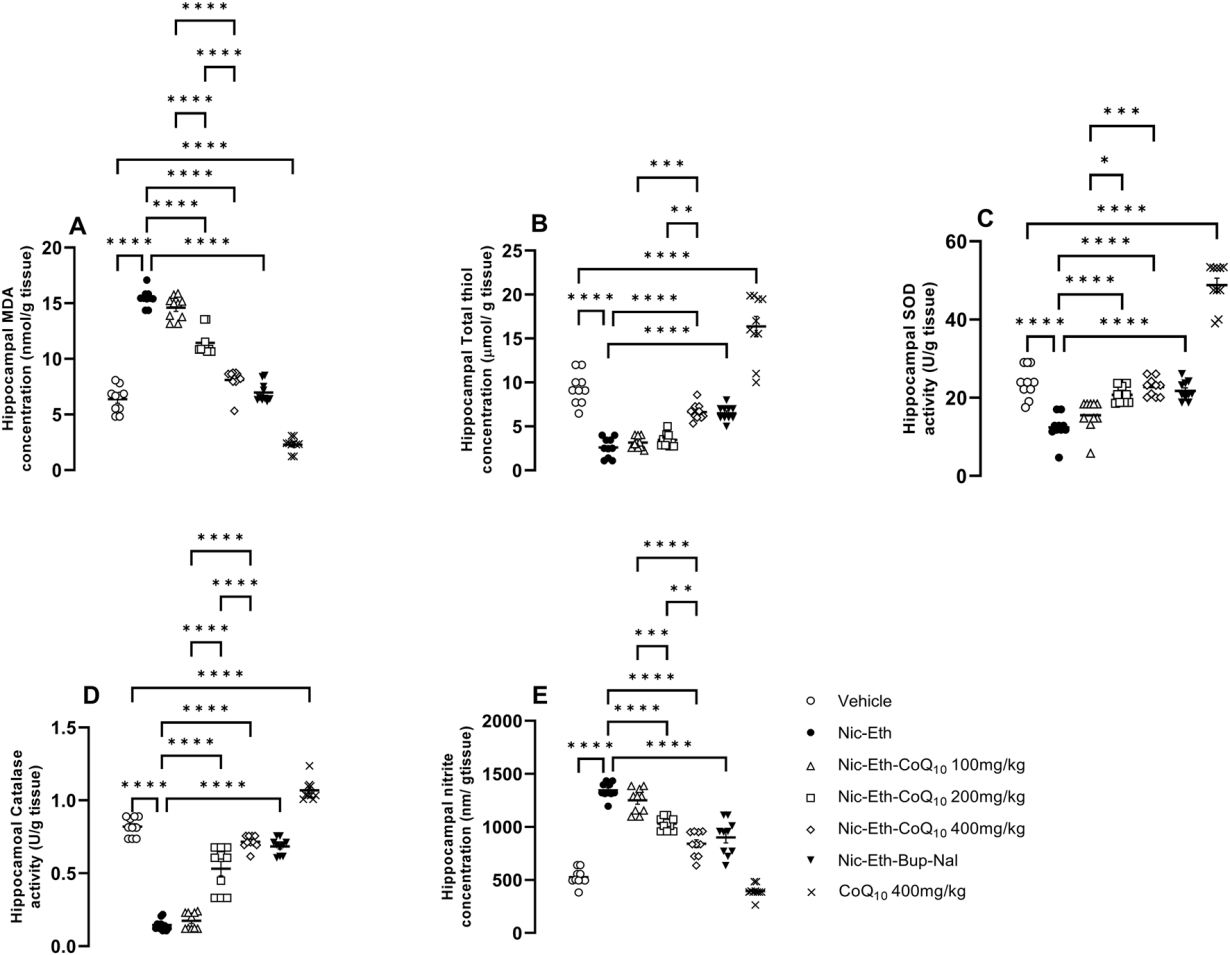
Assessment of oxidative profile in hippocampal tissues. In nicotine-ethanol withdrawn rats, concentrations of pro-oxidant markers including MDA and nitrite was increased and this effect was reversed by CoQ10 treatment (**A** and **E**). On the contrary, anti-oxidant indices including thiol level and SOD/CAT activity were elevated in hippocampi and again this effect was prevented by CoQ10 administration (**B**, **C** and **D**). It should be noted that for all markers, the effect of CoQ10 at its highest dose (400 mg/kg) was almost equipotential to that of the Bup + Nal and the most potent effect was observed in naïve rats received CoQ10 alone (**A–D**). Abbreviations: Nic: nicotine, Eth: ethanol, CoQ10: Coenzyme Q10, Bup: bupropion, Nal: naloxone, MDA: malondialdehyde, SOD: superoxide dismutas. P**** < 0.0001.

Our biochemical results revealed that the hippocampal concentration of pro-oxidant markers including MDA and nitrite increases in rats that experienced a period of Nic-Eth abstinence during adolescence (F (6, 63) = 6.223, *P* < 0.001; Fig. 4A and F (6, 63) = 7.109, *P* < 0.001; Fig. 4E). On the contrary, concentration and activity of anti-oxidant markers including thiol and SOD/CAT decreased, respectively in these animals (F (6, 63) = 11.321, *P* < 0.001; Fig. 4B, F (6, 63) = 5.402, *P* < 0.001; Fig. 4C and F (6, 63) = 21.436, P < 0.001; Fig. 4D). Furthermore, the mentioned changes in both pro-and anti-oxidant parameters were found to be significantly reversed by CoQ_10_ treatment (for MDA 200 and 400 mg/kg, P < 0.01 and P < 0.001 respectively; Fig. 4A, for thiol 400 mg/kg, *P* < 0.001; Fig. 4B, for SOD/catalase/nitrite 200 and 400 mg/kg, *P* < 0.001; Fig. 4C–E). Combination of bupropion and naloxone (Nic-Eth-Bup-Nal group) reversed the effect of Nic-Eth abstinence on all mentioned factors almost with an efficacy similar to that of CoQ_10_ 400 mg/kg (Nic-Eth-Q10 400 group) (P < 0.001; Fig. 4A–E). In this respect, administration of CoQ_10_ 400 mg/kg alone in naïve rats induced the most potent effect (*P* < 0.001; Fig. 4A–E). Dose–response comparisons for the effect of CoQ_10_ were as follows: MDA, (*P* < 0.0001 for 400 mg/kg vs. 100 mg/kg, *P* < 0.0001 for 400 mg/kg vs. 200 mg/kg and *P* < 0.0001 for 200 mg/kg vs. 100 mg/kg). Thiol: (*P* < 0.001 for 400 mg/kg vs. 100 mg/kg and *P* < 0.05 for 400 mg/kg vs. 200 mg/kg). SOD: (*P* < 0.001 for 400 mg/kg vs. 100 mg/kg and *P* < 0.05 for 200 mg/kg vs. 100 mg/kg). CAT: *P* < 0.0001 for 400 mg/kg vs. 100 mg/kg, *P* < 0.0001 for 400 mg/kg vs. 200 mg/kg and *P* < 0.000 for 200 mg/kg vs. 100 mg/kg). Nitrite: *P* < 0.0001 for 400 mg/kg vs. 100 mg/kg, *P* < 0.01 for 400 mg/kg vs. 200 mg/kg, and *P* < 0.001 for 200 mg/kg vs. 100 mg/kg.

### CoQ10 treatment ameliorated the inflammatory response induced by nicotine-ethanol abstinence

**Figure 5:**
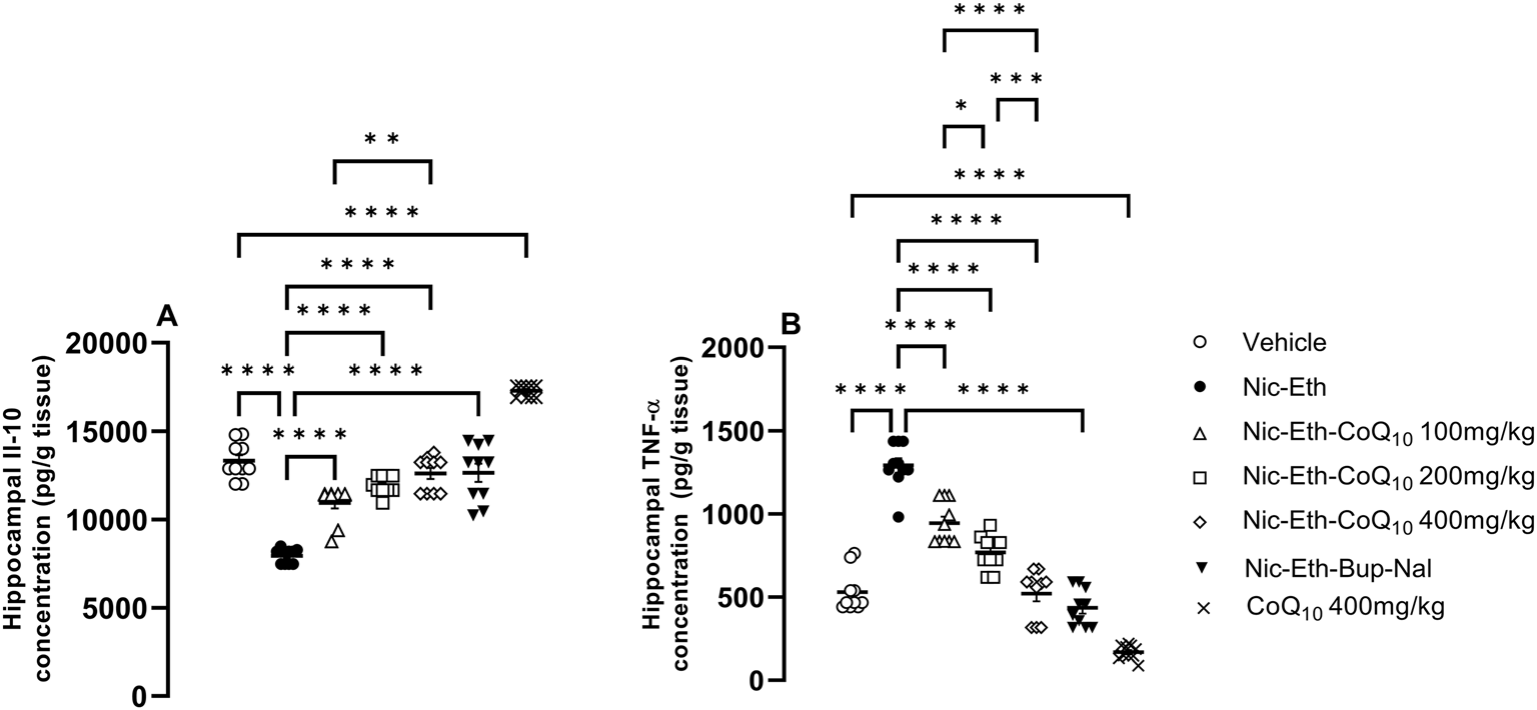
Assessment of inflammatory profile in hippocampal tissues. In nicotine-ethanol withdrawn rats, concentrations of anti- and pro-inflammatory cytokines were decreased and increased including IL-10 and TNF-α, respectively (**A** and **B**). As shown, for both indices, CoQ10 treatment effectively prevented the mentioned effect. In addition, the effect of CoQ10 at the dose of (400 mg/kg) was almost equipotential to that of the Bup + Nal and the most potent effect was found in naïve rats treated by CoQ10 alone. Abbreviations: Nic: nicotine, Eth: ethanol, CoQ10: Coenzyme Q10, Bup: bupropion, Nal: naloxone, IL-10: Interleukin-10 and TNF-α: tumor necrosis factor-alpha. P**** < 0.0001.

It was found that the concentrations of anti-inflammatory cytokine IL-10 reduces in hippocampal tissues of rats undergone Nic-Eth abstinence during adolescence (F (6, 63) = 12.589, *P* < 0.001; Fig. 5A). Conversely, concentration of TNF-α, which is a pro-inflammatory factor, was increased in these animals (F (6, 63) = 9.9, *P* < 0.001; Fig. 5B). As for IL-10 and TNF-α, all doses of CoQ_10_ and bupropion-naloxone combination reversed the effect of Nic-Eth abstinence almost equipotent (*P* < 0.001; Fig. 5A and B). Again, CoQ_10_ 400 mg/kg alone, when administered in naïve rats, induced the most significant anti-inflammatory effect in regard with both IL-10 and TNF-α (*P* < 0.001; Fig. 5A and B, respectively). Dose–response comparisons for the effect of CoQ_10_ were as follows: IL-10: *P* < 0.0001 for 400 mg/kg vs. 100 mg/kg, TNF-α: *P* < 0.0001 for 400 mg/kg vs. 100 mg/kg, *P* < 0.001 for 400 mg/kg vs. 200 mg/kg, *P* < 0.05 for 200 mg/kg vs. 100 mg/kg.

### CoQ10 treatment reversed the effect of nicotine-ethanol abstinence on AChE activity, BDNF and Amyloid-B

**Figure 6:**
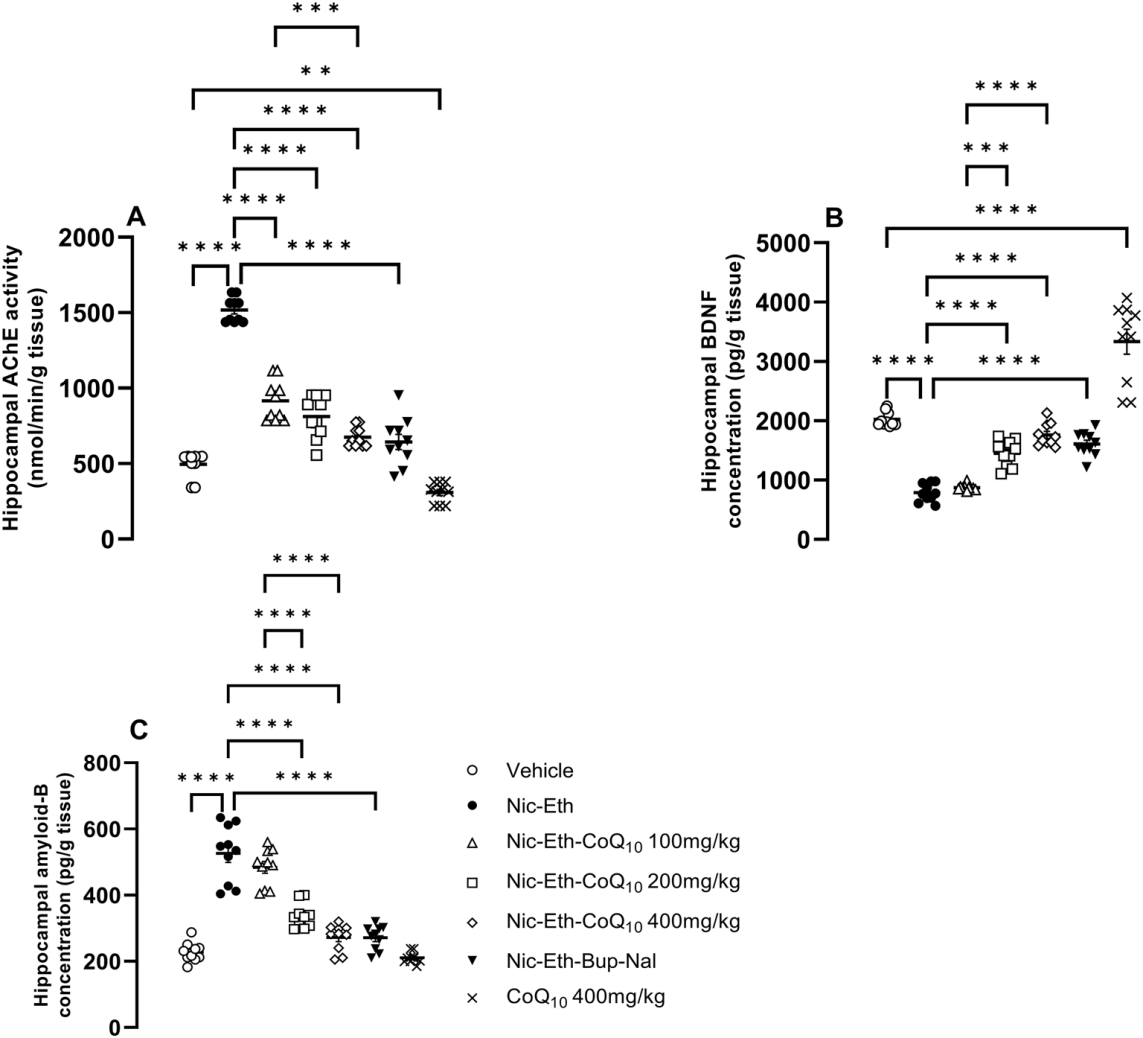
Assessment of AChE, BDNF and amyloid-B in hippocampal tissues. In nicotine-ethanol withdrawn rats, the enzymatic activity of AChE as well as the concentration of amyloid-B was elevated in hippocampal tissues (**A** and **C**), however, BDNF level was diminished significantly (**B**). In all markers, the effect of CoQ10 at the dose of (400 mg/kg) was almost equipotential to that of the Bup + Nal and the most potent effect was found in naïve rats treated by CoQ10 alone. Abbreviations: Nic: nicotine, Eth: ethanol, CoQ10: Coenzyme Q10, Bup: bupropion, Nal: naloxone, AChE: acetylcholinesterase and BDNF: brain-derived neurotrophic factor. P** < 0.01 and P**** < 0.0001.

As shown in Fig. 6, the enzymatic activity of AChE and the concentration of amyloid-B were both increased in hippocampal tissues of rats undergone adolescent Nic-Eth abstinence (F (6, 63) = 22.451, *P* < 0.001; Fig. 6A and F (6, 63) = 8.098, *P* < 0.001; Fig. 6C). On the other hand, tissues level of BDNF was reduced in these animals (F (6, 63) = 18.709, *P* < 0.001; Fig. 6B), however, for AChE, CoQ_10_ treatment at all doses (in Nic-Eth-Q10 group), could effectively reverse the effect of Nic-Eth abstinence (*P* < 0.001; Fig. 6A). Also, in Fig. 6B and C, we showed CoQ10 treatment at two highest doses can reverse the Nic-Eth abstinence effects (P < 0.001; Fig. 6B and C). Combination of bupropion and naloxone induced an improving effect almost similar to that of the highest CoQ_10_ dose (i.e., Nic-Eth-Q10 400 group) (*P *< 0.001; Fig. 6A–C). Finally, administration of CoQ_10_ 400 mg/kg in naïve rats, elevates BDNF and AChE activity in the absence of Nic-Eth treatment. Dose–response comparisons for the effect of CoQ_10_ were as follows: AChE activity: *P* < 0.001 for 400 mg/kg vs. 100 mg/kg, BDNF: *P* < 0.0001 for 400 mg/kg vs. 100 mg/kg and *P* < 0.001 for 200 mg/kg vs. 100 mg/kg. Amyloid-B: *P* < 0.0001 for 400 mg/kg vs. 100 mg/kg and *P* < 0.0001 for 200 mg/kg vs. 100 mg/kg.

## Discussion

In the present study, we first aimed to find out how abstinence from chronic nicotine-ethanol exposure during adolescence could affect the memory function in rats. From clinical point of view, these two phenomena, known as positive and negative reinforcement respectively, promote the individual’s craving for compulsive drug seeking not only to experience euphoria, but also, avoid the undesirable somatic signs. Furthermore, our rationale for co-administration of nicotine and ethanol during adolescence in rats was based on the extensive evidence supporting the strong inclination of human adolescents for tobacco-alcohol co-consumption^54^. For example, majority of individuals with alcohol use disorder also smoke tobacco products regularly^55^. Moreover, chronic smoking has been shown to increase the risk of alcohol use disorder and vice versa^56,57^. It should be noted that the mentioned behavioral link between tobacco smoking and alcohol drinking is primarily established during adolescence^17,18,57^. In other words, not only adolescent alcohol drinkers are more likely to become tobacco smokers, but also adolescent tobacco smokers are more likely to become alcohol drinkers^58^.

In is now well-established that both nicotine and alcohol abstinence following prolonged intake impairs various dysfunctions with a marked adverse effect on learning and memory^33,44,45,59,60^. Studies on human populations have revealed that nicotine-alcohol interaction further complicates the post-quit behavioral outcomes, therefore, finding therapeutic or ameliorative strategies are of paramount significance^61–63^. Up to now, a wide variety of methods have been applied in clinic such as nicotine replacement therapy and pharmacological interventions including bupropion hydrochloride, varenicline, clonidine, monoamine oxidase inhibitors etc., however, efficacy, tolerability and convenience of these options are still the matter of controversy among the researchers^61^.

CoQ_10_ is a critical cofactor in the mitochondrial chain of electron transport and has been found to have neuroprotective, anti-inflammatory, and antioxidant effects in nervous system^64–66^. Consistently, there is clinical evidence indicating that attenuation of cognitive capacities in older adults is associated with reduced plasma concentration of CoQ_10_^67,68^. In line with this evidence, experimental findings have revealed that systemic administration of CoQ10 (10 mg/kg) effectively reverses the impairment of spatial and avoidance memory in rodents^48^. Mechanisms underlying this effect was reported as enhancement of cholinergic activity and improvement of oxidative profile balance in hippocampal tissues^48^. Therefore, in our study, animals were first exposed with simultaneous nicotine-ethanol administration and then received CoQ10 within the “abstinence period” i.e., following cessation of drugs. CoQ_10_ was orally administered in order to mimic the supplementation applicability in clinical practice. In addition, three escalating doses of CoQ_10_ was applied to test the dose-dependent nature of drug effects at behavioral level. Results indicated that abstinence from nicotine-ethanol exposure markedly impairs the development of memory in rats when tested by MWM and NORT, respectively (Figs. 2 and 3). This adverse effect was characterized by reduced slope of the curve in Fig. 2A, representing the fact that development of learning is delayed in rats. In other words, animals experienced nicotine-ethanol abstinence, leases rapidly learn and remember how to find the escape platform in water tank. In line with this, these subject spend a considerably less swimming time within the target quadrant in which the escape platform is located. Our biochemical tests revealed that these behavioral deficits most probably result from increased oxidative markers, decreased anti-oxidant and neurotrophic capacity, exacerbation of inflammatory response, reduced cholinergic activity and accumulation of amyloid-B in hippocampal tissues. Interestingly all behavioral outcomes as well as the mentioned biochemical alterations were significantly reversed in animals treated by CoQ_10_ and in most cases the ameliorative effect was found to be dose-dependent (Figs. 4, 5 and 6). Consistent with these findings, the somatic signs of nicotine-alcohol abstinence were also decreased in animals treated by CoQ_10._ This particularly matters in clinical context because the individual treated by CoQ_10_ is protected from memory dysfunction, while he/she experiences less physical suffering (negative reinforcement). Aside from these, our study included an independent group in which the subjects received bupropion and naloxone simultaneously during the abstinence interval (i.e., following cessation of nicotine and alcohol). The inclusion of this experimental group was done to find out whether the efficacy of CoQ_10_ is comparable to that of bupropion and naloxone (both of which are commonly used in clinic for the management of smoking and alcohol abstinence syndromes)^69–74^. What we observed in our findings was truly intriguing because in all experiments, CoQ_10_ treatment at the dose of 400 mg/kg induced almost the same effect as obtained by bupropion-naloxone co-administration. Therefore, it seems reasonable to suggest the use of CoQ_10_ instead or in combination with bupropion and/or naloxone to ameliorate the adverse effects of nicotine-alcohol abstinence.

Our rationale for choosing these specific biochemical markers, was based on the results of our recent studies in which we observed that both nicotine and alcohol abstinence could induce memory deficits through alteration of oxidative status, inflammatory response, cholinergic activity, BDNF and amyloid-B in hippocampal tissues of adolescent male rats^33,44,45,52^. In addition, there is a mechanistic connection between cholinergic function and inflammatory response, known as “cholinergic anti-inflammatory pathway” in literature. In brief, release of inflammatory cytokines (secondary to tissue damage, infection, ethanol/nicotine abstinence etc.) activates the vagus nerves ascending to the brain stem which is then excites the efferent vagus nerves descending to the visceral organs. Then, Ach release via cholinergic terminal promotes the suppression of further cytokine secretion by macrophages through activation of α7-nicotinic acetylcholine receptors^75^.

Therefore, in our results, elevation of acetylcholinesterase activity by nicotine-ethanol abstinence (in Fig. 6A) causes more Ach degradation in synaptic cleft and this attenuates the mentioned function of cholinergic pathway, as characterized in our results by increased level of TNF-a (Fig. 5B). On the other hand, the anti-inflammatory effect of CoQ10 (characterized by reduction of TNF-a, Fig. 5B) could be explained by potentiation of the “cholinergic anti-inflammatory pathway”. As we observed (in Fig. 6A), CoQ10 reduced the activity of acetylcholinesterase which represents more Ach availability in synaptic cleft to bind and activate the α7-Ach receptors.

Although the neuroprotective effects of CoQ_10_ is rather established in literature, this issue has not been addressed in association with nicotine/alcohol abstinence in humans. Moreover, the present study, for the first time, probed into the neurobiological mechanism of CoQ_10_ in this context. Clinical trials are essential to reveal whether prolonged and high dose administration of CoQ_10_ is safely tolerated by nicotine and/or alcohol-dependent patients. It should be noted that since we did not measure the behavioral characteristics of immature brain function in rats (as expected in adolescents such as impulsivity, risk taking etc.), one should carefully compare/generalize our findings to the evidence obtained from studies on adolescent brains in human subjects. In this regard, the use of “early-life” (instead adolescent) drug exposure may represent a more accurate term. Finally, the present study included only male subjects both in design and experimentations. This particularly matters in respect with the aforementioned sex-dependent discrepancies in craving for drug abuse among adolescents^40^. Moreover, recent studies on animal models have revealed that such differential reward behaviors are associated with differential function of nicotinic acetylcholine receptor system^76^.

## Conclusion

According to the results of this study, abstinence from adolescent nicotine and alcohol exposure results in impairment of memory function. These adverse effects were found to be mediated by changes in the level/activity of oxidative, inflammatory, neurotrophic and cholinergic markers within the hippocampi. Moreover, administration of CoQ_10_ could effectively prevent the frequency of abnormal signs and memory deficits at behavioral level as well as the mentioned biochemical alterations. As for the involved mechanisms, it seems that abstinence from nicotine and ethanol causes the neuroinflammation in hippocampal tissues and it might even exacerbate this phenomenon by suppression of cholinergic anti-inflammatory pathway. This could occur secondary to elevation of acetylcholinesterase and subsequent reduction of synaptic Ach levels. The opposite scenario explains how CoQ_10_ induces its anti-inflammatory effects, i.e., probably through potentiation of cholinergic anti-inflammatory mechanism. However, this hypothesis has never been proposed or investigated within the hippocampal region and requires precise investigation in future studies.

## Materials and methods

### Animals, drugs and experimental protocols

Male Wistar rats (postnatal day 21–42, weighing 50 ± 5 g) were obtained from the animal house at Torbat Heydariyeh University of Medical Sciences, Iran (in-house breeding facility). Animals were kept in Plexiglas cages with ad libitum access to food and water in a colony room under constant temperature (22 ± 2 ◦C) and 12-h light/dark cycle (Lights on at 6:00 AM).

All methods were carried out according to the relevant guidelines and regulations (Ethics number issued by ethics committee of Torbat Heydariyeh University of Medical Sciences: IR.THUMS.AEC.1402.002). In addition, all methods were reported in accordance with ARRIVE guidelines^77^. Attempt was made to minimize the suffering and the number of animals used in this study. Ethanol 99.9% were purchased form Dr. Mojallali Co. and freshly prepared prior to the experiments. Based on the methods of previous^44,78^ studies, an escalating protocol was applied for addition of ethanol to animals’ drinking water as follows: 5% (PNDs 21–22), 10% (PNDs 23–24), 15% (PNDs 25–26) and 20% (PNDs 27–42). Nicotine, as (-)-Nicotin, C_10_H_14_N_2_, Merck, Germany) was obtained from Merck Co. and injected i.p. at the dose of 2 mg/kg. CoQ_10_ and bupropion were administered through oral gavage. Naloxone was injected i.p. at the dose of 10 mg/kg. All daily drug administrations, including injection and gavage, were done at 8:00 am by the same experimenter who was not informed of the study design.

Subjects were randomly assigned to 7 experimental groups (n = 10 in each group), as follows: Group 1: Animals were intraperitoneally (i.p.) injected with saline (NaCl 0.9%) in their adolescence (PND 21–42) and then received saline again through oral gavage for three weeks to undergo abstinence (PND 43–63). Group 2: animals received co-administration of nicotine (2 mg/kg i.p) and ethanol (in their drinking water) during adolescence (Nic-Eth group, PND 21–42) and then received saline through oral gavage for three weeks to undergo abstinence (PND 43–63). Groups 3–5: animals received co-administration of nicotine (2 mg/kg i.p) and ethanol (in their drinking water) during adolescence (PND 21–42) and then received CoQ_10_ through oral gavage for three weeks (Nic-Eth- CoQ_10_ group, PND 43–63) at three escalating doses of 100, 200 and 400 mg/kg. Groups 6: animals received co-administration of nicotine (2 mg/kg i.p) and ethanol (in their drinking water) during adolescence (PND 21–42) and then received co-administration of bupropion (20 mg/kg, i.p.) and naloxone (10 mg/kg, i.p.) for three weeks (PND 43–63). Groups 7: Animals were injected with saline (i.p.) in their adolescence (PND 21–42) and then received the highest dose of CoQ_10_ (400 mg/kg) through oral gavage for three weeks (PND 43–63). It should be noted that the exposure timeframe (i.e., PND 21–42) in our study actually covers prepubescent (or juvenile) to mid adolescents in rats^79–81^. Behavioral tests including the Morris water maze (MWM) and novel object recognition (NOR) test were performed between PNDs 63–69. Then, animals were first euthanized by carbon dioxide inhalation, then rapid decapitation was done using a guillotine. The skull was gently removed to access and excise the whole brain. In the next step, both hippocampi were carefully dissected under stereoscope by an expert researcher. Then, tissue samples were taken and transferred to microtubes to be kept frozen at -80 °C until use for biochemical measurements (see timeline in Fig. 1).

### Behavioral assessments

#### MWM test

The MWM, has become as one of the most common behavioral tests for assessing memory function alongside spatial memory in rodents. The device is consisted of a large circular pool equipped with the hidden platform. The test includes 6 days in which five consecutive days are considered as trial period and the 6th day is known as the test phase. Each day consists of 4 trials and in each trial the animals are released into water from a fixed location and allowed to swim and find the escape platform within 60 s. The time spent to find the hidden platform was recorded and processed. An inter-trial interval of 20 s was considered for all subjects. As expected, the more memory disturbance leads to the more time spent to reach the platform. Eventually, on probe day (sixth day), the escape platform was removed to assess spatial memory and how animals remember the quadrant in which the hidden platform was submerged. In this regard, they were allowed to swim and find the time spent in the desirable quadrant (the hidden platform was located). In fact, the more memory impairment leads to the less time spent in the desirable quadrant.

#### NOR test

The NOR test, was used to assess the memory function in rats based on the methods previously established in literature^82,83^. In this methods, the test box includes an open square field (45 × 45 × 50 cm^3^) in which animals undergo three trials of habituation, training and testing. On day 1, rats are allowed to freely explore the filed for 5 min and on day 2, they have the opportunity to explore two identical objects in the box through touching and nose-poking. On day 3 (test day), the experimenter replaces one of the objects by a new one and animals can explore both familiar and novel objects for 5 min. In order to quantify the memory function in subjects, a discrimination index is calculated via the following formula: “Time of novel object exploration” divided by “Total exploration time” × 100.

#### Assessment of physical manifestations during nicotine-ethanol abstinence

In order to assess the effect of nicotine-ethanol abstinence (PNDs 42–63) on physical manifestations among different experimental groups, animals were put in clear Plexiglas cages to visually check how many of them per group display specific somatic signs including tremor, eye blink, yawning and grooming during 15 min. At the end of assessment, animals were returned to their home cages.

### Biochemical assessments

In order to reveal what cellular mechanisms mediate our behavioral observations, the following biochemical parameters were measured in hippocampal tissues: 1. Pro/antioxidant markers including superoxide dismutase (SOD), catalase, total thiol, nitrite and malondialdehyde (MDA). In addition, the inflammatory response was assessed via quantification of Interleukin-10 (IL-10) and tumor necrosis factor-alpha (TNF-α). Finally, acetylcholinesterase (AChE) activity plus the concentration of brain-derived neurotrophic factor (BDNF) and amyloid-B and were measured.

#### Blood content of ethanol, MDA, Thiol, Nitrite, SOD and catalase

At the end of nicotine-ethanol exposure, blood samples were taken and ethanol content was measured as mg/dl by Cobas Integra 400 plus analyzer (Roche Diagnostic GmbH, Germany). The MDA tissue concentration, as a typical marker of lipid peroxidation, was measured using a previously described method. In brief, MDA reacts with thiobarbituric acid (TBA) and produces a red substance. In case of thiol, there is a reaction between DTNB (2, 2′ -dinitro-5,5′ -dithiolbenzoic acid) and thiol groups which finally forms a yellow complex. In both cases, absorbance level of the colorful final products are read by a spectrophotometer. For measurement of SOD and catalase activities, Madesh and Aebi protocols were used, respectively^84,85^. In brief, the activity of SOD is quantified through a colorimetric method with a detection threshold at 570 nm and as for the CAT activity, hydrogen peroxide acts as a substrate in the reaction. In addition, hippocampal concentration of nitrite was measured by a commercial colorimetric assay kit (Promega Corporation, USA). In this method, the supernatant of homogenized samples react with the Griess reagent and the absorbance is recorded at 520 nm.

#### Concentrations of IL-10 and TNF-α, BDNF, Amyloid-B and AChE

In order to measure the hippocampal concentrations of IL-10, TNF-α, BDNF and amyloid-B, a commercial rat ELISA kits was purchased and the manufacturer's instructions were followed (IBL International, Hamburg, Germany, and MyBioSource, San Diego, CA, USA). A microplate reader (Biotech, USA) was used to measure the absorption rates and results were compared to the standard curve in the same experiment. The activity AChE enzyme was measured by a method described in previously^86^. In brief, the reaction proceeds between the thiol group of acetylthiocholine iodide as a substrate and the 5,5-dithiobis-2-nitrobenzoate ion which promotes the synthesis of 5-thio-2- nitrobenzoic acid. The absorbance rate is read at the wavelength of 405 nm and the enzymatic activity is obtained in comparison with the standard curve.

### Statistical analyses

Data were transferred to GraphPad Prism software (version 6, USA) for statistical analyses. Two-way ANOVA test was applied for the analysis of MWM test, in which two independent variables (i.e., Time and Day) were addressed. For other results (NOR and biochemical tests), in which only one independent variable was the subject of analysis (i.e., time or concentration), one-way ANOVA was applied followed by Tukey's post hoc test. Fisher's exact test was used to analyze the effect of nicotine-ethanol abstinence. In all analyses, *P*-value < 0.05 was considered statistically significant and data were presented as mean ± standard error of the mean (SEM).

## Data Availability

The datasets generated during and/or analyzed during the present study are available from the corresponding author.
